# Role of miR-191/425 Cluster in Tumorigenesis and Diagnosis of Gastric Cancer

**DOI:** 10.3390/ijms15034031

**Published:** 2014-03-05

**Authors:** Wei-Zhao Peng, Ren Ma, Fang Wang, Jia Yu, Zhi-Bin Liu

**Affiliations:** 1 Department of General Surgery, China-Japan Friendship Hospital, Beijing 100029, China; E-Mails: pwz_75@163.com (W.-Z.P.); maren517@sohu.com (R.M.); 2 Department of Biochemistry and Molecular Biology, Institute of Basic Medical Sciences, Chinese Academy of Medical Sciences and Peking Union Medical College, Beijing 100005, China; E-Mail: wo_wfang@hotmail.com

**Keywords:** miR-191, miR-425, serum miRNA, gastric cancer

## Abstract

Gastric cancer (GC) is among the most frequent types of cancer worldwide. Therefore, understanding the biology of GC tumorigenesis is important for appropriate diagnosis and patient surveillance. The miR-191/425 cluster has been reported to be overexpressed in various human cancers, but the tumorigenic role and clinical significance of miR-191/425 overexpression in gastric carcinogenesis is currently undefined. In this study, the expression of miR-191 and miR-425 in GC tissue and serum was assessed, and the relationship between miRNA expression and clinicopathological data was analyzed. We found that miR-191 and miR-425 were both significantly increased in human GC tissues relative to adjacent normal controls. In addition, miR-191 levels correlated with GC tumor stage and metastatic state. Furthermore, the level of serum miR-191 was significantly higher in the GC group than in the control group when using serum miR-16 as an endogenous control. Finally, inhibition of miR-191 or miR-425 in the GC cell lines HGC-27 not only reduced cell proliferation and cell cycle progression but also impaired cell migration and invasion. Taken together, our results revealed the oncogenic roles of miR-191 and miR-425 in gastric carcinogenesis, and indicated the potential use of serum miR-191 as a novel and stable biomarker for GC diagnosis.

## 1. Introduction

Cancer accounts for the highest mortality in developed countries and the second highest in developing countries, making it a worldwide health problem [1,2]. Gastric cancer (GC) is among the most frequent types of cancer worldwide, accounting for approximately 8% of new malignancies every year [3,4]. Among the incidence of cancers worldwide, GC ranks fourth in males and fifth in females, while the mortality rate ranks third in males and fifth in females [5]. Hence, the prediction of the biology and course of GC tumorigenesis is important for appropriate diagnosis and patient surveillance.

MicroRNAs (miRNAs) are a class of evolutionarily conserved non-coding RNAs that pleiotropically suppress expression of protein-coding genes at the post-transcriptional level [6–8]. The spectrum of miRNAs expressed in tumor cells differs dramatically from normal cells and it is now well established that miRNAs play key roles in essentially all aspects of tumor biology [8]. miRNAs have been shown to be divergently expressed in GC neoplastic tissues relative to normal tissues [9,10] and to play important roles in GC initiation and progression [10]. For example, transfection of miR-181b into GC cells has been shown to significantly increase cell proliferation, migration and invasion [11]. miR-7 was reported to function as an anti-metastatic miRNA in GC by targeting the insulin-like growth factor-1 receptor [12]. Therefore, targeting the miR-7/IGF1R/Snail axis may be useful as a therapeutic approach for blocking GC metastasis [12]. The downregulation of prohibitin by miR-27a may explain why the suppression of miR-27a inhibits GC cell growth [13]. Lastly, miR-15b and miR-16 are able to modulate the sensitivity of GC cells to certain anticancer drugs, at least in part by regulating BCL2 expression [14].

Methods to reliably detect miRNA in the circulatory system of GC patients have also been demonstrated. For example, the levels of miR-21 and miR-223 in plasma samples of GC patients were demonstrated to be significantly higher than in healthy controls, while miR-218 was significantly lower [15,16]. In addition, miR-223 was specifically correlated to *Helicobacter pylori* infection, an etiological agent for GC [17]. Other miRNAs proposed as diagnostic markers for GC include miR-151-5p and miR-199a-3p, miR-221, miR-200c and miR-378 [18–21]. As circulating miRNA levels are closely associated with the tumorigenesis, progression and prognosis of GC, the detection of miRNAs in the circulatory system may offer new biomarkers for GC.

miR-191 is associated with several human solid tumors including colon, lung, pancreas, prostate, and stomach cancer, as well as acute lymphocytic leukemia (ALL)-associated hematopoietic malignancies [22–24]. miR-191 was hypomethylated and overexpressed in liver cancer, and the inhibition of miR-191 decreased cell proliferation and tumor growth of hepatocellular carcinoma cells [22]. The activation of miR-191 and miR-425 (the miR-191/425 cluster) expression by their host gene DALRD3 and estrogen receptor a (ERa) was shown to modulate the tumorigenicity of breast cancer cells [24]. miR-191 also displayed tumor-type specific roles in tumorigenesis, as miR-191 represses MDM4 and CDK6 expression in ovarian and thyroid follicular cancer, thereby delaying cancer progression and tumor-related death [25,26]. However, the precise role for the miR-191/425 cluster and their clinic significance in human GC are not well understood.

In the present study, we focused on the tumorigenic role of the miR-191/425 cluster in human GC cells as well as their potential use for the diagnosis of GC. Our results may provide new insight into the role of the miR-191/425 cluster in human GC tumorigenesis and may suggest serum miRNA levels to be novel clinical biomarkers in GC patients.

## 2. Results and Discussion

### 2.1. The miR-191/425 Cluster Is Highly Expressed in Gastric Cancer (GC)

**Figure 1.**
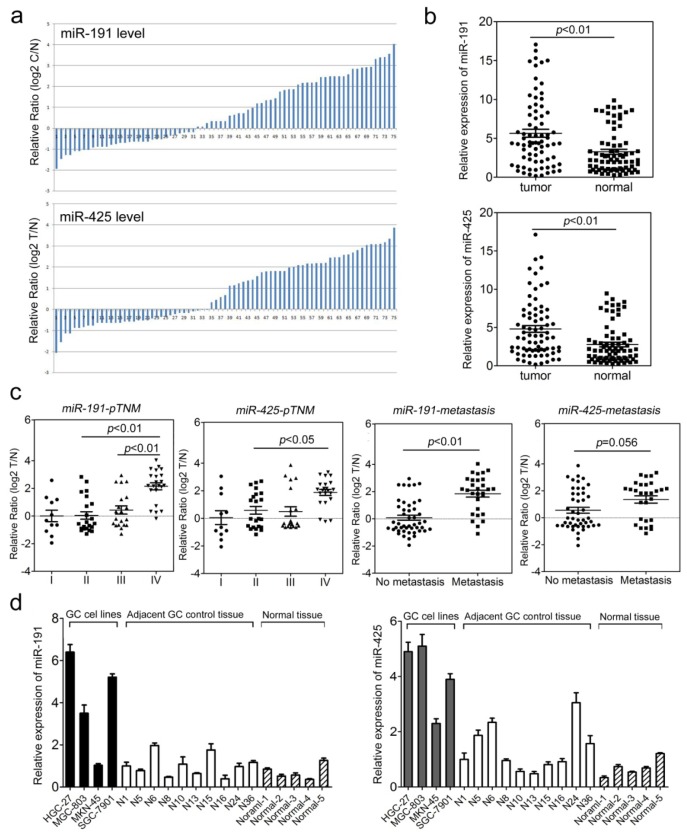
Expression of miR-191 and miR-425 in gastric cancer (GC) tissues and cell lines. (**a**) miR-191 and miR-425 were detected in 75 GC tissue samples with the adjacent normal controls by quantitative RT-PCR. Data are presented as log2 of fold change of GC tissues relative to adjacent normal regions; (**b**) Relative miR-191 and miR-425 expression levels in GC tissues and adjacent normal regions; (**c**) Statistical analysis of the association between miRNA levels and the pTNM stage (I, II, III and IV) and pM stage (No metastasis and Metastasis). Analysis of variance (ANOVA) was used to determine whether there were any significant differences between groups; (**d**) Relative levels of miR-191 and miR-425 in GC cell lines (HGC-27, MGC-803, MKN-45 and SGC-7901) relative to 10 GC adjacent normal control samples (Adjacent GC control tissue) and five normal gastric tissue biopsies from healthy people (Normal tissue).

The fact that miRNAs originating from the same cluster can reinforce their action on common cellular pathways led us to investigate the association of the miR-191/425 cluster with gastric carcinogenesis. To determine the differential expression of miR-191 and miR-425, we used quantitative RT-PCR (qPCR) to analyze their relative levels in total RNA extracted from 75 clinical GC tissue samples with adjacent normal tissue samples from the same patient. The expression of miR-191 and miR-425 was much higher in tumor than in non-tumor tissues (Figure 1a,b). To further study the correlation between miRNA expression and clinicopathological factors, the levels of miR-191/425 in GC tissues (including fully clinical information) were statistically analyzed (non-parametric test). The level of miR-191 was significantly higher in GC patients with late stage disease (*p* < 0.01, stage IV *vs.* stage II; *p* < 0.05, stage IV *vs.* stage III) (Figure 1c). A similar trend was observed in miR-425 expression among patients with stage IV disease compared to stage II disease (*p* < 0.05, stage IV *vs.* stage II) but not stage III disease (*p* = 0.065) (Figure 1c). We next investigated whether miRNA levels were affected by tumor metastasis. The level of miR-191 in GC tissues with metastasis was higher than that in the non-metastatic group (*p* < 0.01). However, the miR-425 level was similar in patients with different pM stages (*p* = 0.056) (Figure 1c). Examination of miR-191 and miR-425 expression in four GC cell lines (HGC-27, MGC-803, MKN-45 and SGC-7901) indicated an obvious upregulation of miR-191/425 in HGC-27, MGC-803, and SGC-7901 cell lines when compared to adjacent non-tumor tissues and five normal gastric tissues from healthy people (Figure 1d). These results, particularly the finding that high levels of miRNAs correlate with disease progression, indicate the misregulation of miR-191 and miR-425 in GC patients, which makes the use of miR-191 and miR-425 as novel biomarkers in GC patients a possibility.

### 2.2. Clinical and Pathological Characteristics of miR-191/425 Levels in GC Serum

**Figure 2.**
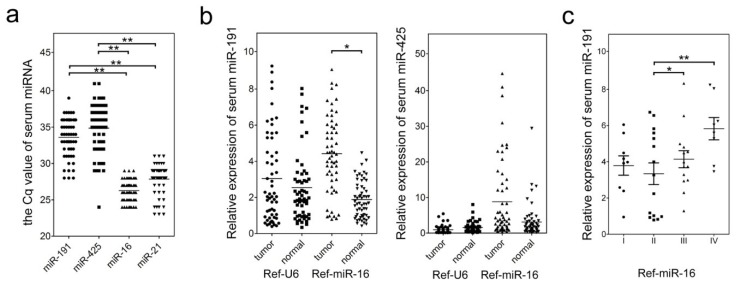
Comparison of serum miRNAs between GC patients (tumor) and healthy controls (normal). (**a**) Raw quantification cycle (*C*q) data for miR-191 and miR-425 in the control blood samples (*n* = 57) are depicted; (**b**) Changes of serum miRNAs between the GC patients (tumor) and the healthy control (normal); U6 snRNA and miR-16 were used as the endogenous control for data normalization, respectively; (**c**) Statistical analysis of the association between serum miR-191 level and pTNM stage (I, II, III and IV); miR-16 was used as the endogenous control for data normalization. Analysis of variance (ANOVA) was used to determine whether there were any significant differences between groups. * *p* < 0.05; ** *p* < 0.01.

To determine the level of circulating miR-191/425 level in GC patient serum, we performed qPCR analysis and compared miR-191/425 expression in GC patient serum with normal human serum (a control group consisting of pooled RNA from 58 healthy donor serum samples). First, we detected the raw *C*q data for miR-191 and miR-425 in the control serum. We used previously reported levels of circulating miR-21 with clinical significance in serum of different solid tumors (including breast cancer, esophageal cancer, gastric cancer, colorectal cancer and lung cancer) as a positive control [27]. miR-16, the serum level of which has been shown to have no significant difference between GC samples and normal controls [28], was used as a negative control. In the normal serum, the mean *C*q of both miR-191 (*C*q = 33) and miR-425 (*C*q = 35) was higher than the mean *C*q of both miR-16 (*C*q = 26) and miR-21 (*C*q = 28) (Figure 2a). This difference was significant (Figure 2a), suggesting a lower background in non-tumor serum for miR-191/425 expression relative to miR-16 and miR-21. Furthermore, the low basal levels of serum miR-191 and miR-425 might hint that miR-191/425 could serve as more sensitive biomarkers in GC detection.

To ascertain whether the miR-191 and miR-425 signatures differ between GC and non-tumor serum, qRT-PCR was performed using 57 GC patients and 58 healthy donor blood samples. When U6 snRNA was used as an endogenous control for data normalization, no miRNA levels were significantly different between the two groups (*p* = 0.23, miR-191; *p* = 0.07, miR-425) (Figure 2b). In contrast, when using miR-16 as an endogenous control, the relative level of miR-191 in GC serum was significantly higher than those in the controls (*p* < 0.01) (Figure 2b). Subsequently, we evaluated whether there was a correlation between the level of miR-191 and clinical characteristics of patients. These results indicate that increased levels of serum miR-191 in GC samples correlate with pTNM stage (*p* = 0.013, stage II *vs.* III; *p* = 0.009, stage II *vs.* IV) (Figure 2c) but not with other clinicopathologic features.

### 2.3. Evaluation of Serum miR-191/425 as a Potential GC Diagnostic Marker

**Figure 3.**
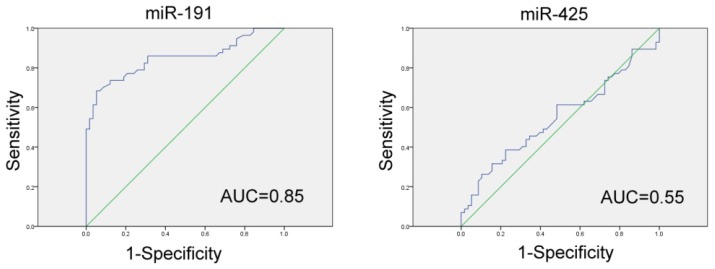
Receiver–operator characteristic (ROC) curve analysis using serum miRNAs for discriminating the GC group from the controls. Abbreviation: AUC, area under the ROC curve.

To evaluate whether serum miR-191 and miR-425 levels can be used as potential diagnostic markers for GC, ROC curve analyses were performed. Levels of serum miR-191 can be a potential marker for discriminating GC patients from healthy controls, with ROC curve areas of 0.849 (95% CI: 0.78–0.92) (Figure 3). However, serum miR-425 levels are not suitable for GC diagnosis (ROC curve areas of 0.548 (95% CI: 0.44–0.65)) (Figure 3). According to the ROC curve, the relative blood level of miR-191 is 3.34, which is the optimal cutoff value for differentiating GC patients and controls (Youden’s index). With this cutoff value for miR-191, the sensitivity and specificity were 70.2% and 99.9%, respectively. The relative expression values for miR-191 in blood above this cutoff point were found in 50.0% of stage I–II GC patients, in 71.4% of stage III GC patients and in 75.0% of stage IV GC patients. These findings suggest that elevated blood miR-191 could be detected in early stages of GC and therefore facilitate early disease detection.

### 2.4. Inhibition of miR-191 and miR-425 Repressed Cell Proliferation and Cell Cycle Progression of GC Cells

**Figure 4.**
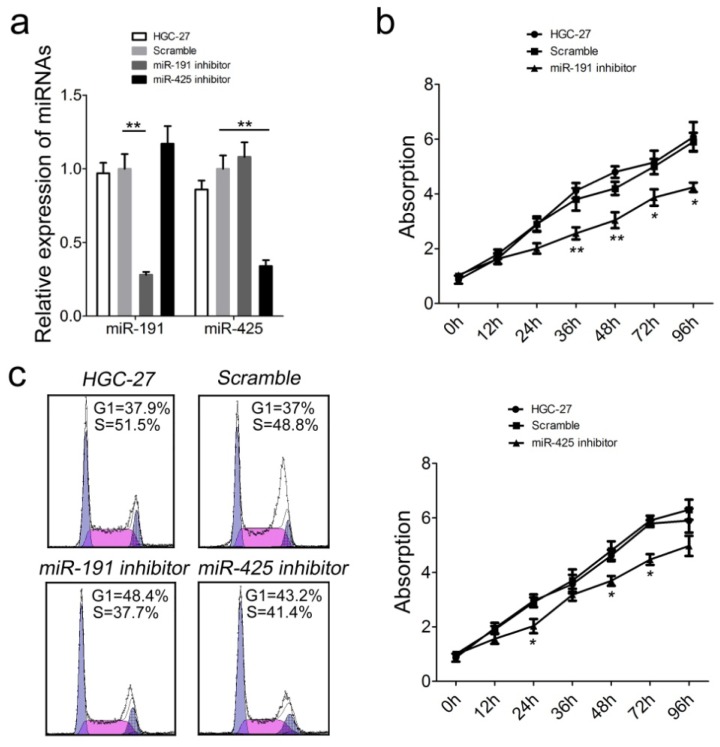
The influence of miR-191 and miR-425 expression on GC cell proliferation and cell cycle progression. (**a**) MiR-191 and miR-425 levels were detected by quantitative RT-PCR in HGC-27 cells after treatment with miRNA inhibitor (25 nM) or scramble control (25 nM); (**b**) CCK-8 cell proliferation assay of HGC-27 cells after treatment with miRNA inhibitor or scramble control; (**c**) Cell cycle analysis of HGC-27 cells after treatment with miRNA inhibitor or scramble control by propidium iodide (PI) staining. Error bars represent the standard deviation obtained from three independent experiments. * *p* < 0.05; ** *p* < 0.01.

The remarkable increase in miR-191 and miR-425 expression in GC patients incited us to explore the possible biological significance of these miRNAs in tumorigenesis. To assess the relevance of miR-191/425 levels on GC cell growth, miRNA inhibitors or scramble controls were used to transfect HGC-27 cells. Treatment with miRNA inhibitors reduced intracellular levels of miR-191 and miR-425 in HGC-27 cells by 4-and 3-fold over scramble control, respectively (Figure 4a). Proliferation of transfected HGC-27 cells was measured using a CCK-8 assay. Reduced expression of miR-191 and miR-425 led to significant decreases in cell proliferation in HGC-27 cells (Figure 4b). Accordingly, the percentage of S phase cells was reduced by ~11% and ~8% in HGC-27 cells treated with miR-191 and miR-425 inhibitor, respectively (Figure 4c). Taken together, these results indicate that inhibition of miR-191 and miR-425 can efficiently reduce tumor cell proliferation and cell cycle *in vitro*, suggesting oncogenic roles in modulating tumorigenicity of GC cells.

### 2.5. Inhibition of miR-191 and miR-1425 in GC Cells Inhibits Cell Migration and Invasion

**Figure 5.**
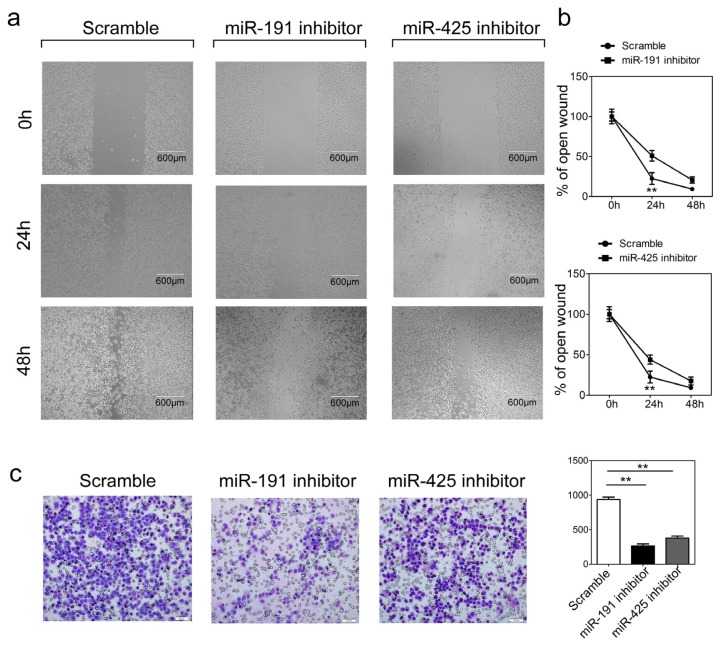
The influence of miR-191 and miR-425 expression on GC cell migration and invasion. (**a**,**b**) Wound healing assays of HGC-27 cells after treatment with miRNA inhibitor or scramble control. Relative ratios of wound closures per field is shown in (**b**); (**c**) Transwell analysis of HGC-27 cells after treatment with miRNA inhibitor or scramble control. The relative ratios of invasive cells per field are shown. Scale bar: 50 μm. Error bars represent the standard deviation obtained from three independent experiments. ** *p* < 0.01.

Rapid invasion and metastasis of tumor cells are responsible for poor prognosis and the major cause of death in GC patients. Based on our results that miR-191 or miR-425 are statistically associated with metastasis and the degree of tumor malignancy, we proposed that these two miRNAs might play an extremely important role in GC cell migration and invasion. To test our hypothesis, cell migration and invasion assays were performed in HGC-27 cells transfected with miR-191/425 inhibitors or scramble controls. Inhibition of endogenous miR-191 and miR-425 in HGC-27 cells resulted in a significant reduction of cell migration during the closing of artificial wounds created over confluent monolayers (Figure 5a,b). HGC-27 cells were maintained in serum-free medium during the course of wound healing to ensure that any augmented migratory behavior could not be affected by altered cell proliferation. In addition, reduced expression of miR-191 and miR-425 dramatically inhibited the normally strong invasive capacity of HGC-27 cells as indicated by transwell invasion assays (Figure 5c). These results are consistent with the above findings that the miR-191 and miR-425 can promote tumor cell growth as well as their progression towards more malignant degree.

### 2.6. Discussion

Recently, the search for novel biomarkers for diagnostic tools is one of the most rapidly growing areas in cancer research. Blood-based protein biomarkers, such as carcinoembryonic antigen (CEA), carbohydrate antigen (CA), or prostate specific antigen (PSA), have gained much recognition in the past decades [29]. However, they suffer from low sensitivity, especially with respect to their inability to screen for early stages or to distinguish aggressive from indolent tumors. Therefore, the discovery of circulating miRNAs present in cell-free body fluids such as plasma, serum, urine and saliva represent a new approach for the diagnostic screening of tumors. miRNAs were first found to be elevated in the serum of lymphoma patients compared to healthy individuals [30]. Since then, circulating miRNAs have attracted a great deal of attention as novel, minimally invasive biomarkers for various cancers. GC has one of the poorest survival rates among various types of cancers, which makes the discovery of suitable biomarkers for early detection or prognosis all the more important [2]. Previous studies have revealed some circulating miRNAs with diagnostic significance in GC patients, such as miR-17-5p, miR-21, miR-106a, miR-106b, and let-7a [15,17]. The first four miRNAs were shown to be present at significantly higher levels in GC plasma samples, whereas let-7a was lower in GC patients than in healthy individuals. Another study delineated miR-221, miR-376c, and miR-744 as markers for GC detection, which were capable of identifying GC even 5 years prior to clinical diagnosis [28]. miR-223 was also shown to be significantly higher in GC plasma samples than in healthy controls. Interestingly, miR-223 levels also correlated to *Helicobacter pylori* (*H. pylori*) infection, an etiological agent for GC [17]. Our results revealed that serum miR-191 level was significantly increased in GC patients compared to controls. Because the basal level of miR-191 in non-tumor serum is relatively low, even a small increase in its expression could enable a sensitive enough signal for detection. Furthermore, serum miR-191 levels were also associated with lymph node metastasis and TNM staging, thus increasing its diagnostic importance.

Currently, qRT-PCR is the most frequently used approach for quantitative analysis of circulating miRNAs. Therefore, normalization is an essential step of reliable qRT-PCR results because different reference genes would yield different outputs. A recent study comparing serum levels of 24 miRNAs in GC patients before and 1 year after *H. pylori* eradication drew different conclusions when different reference genes were used [31]. In this study, none of the miRNAs were significantly different between the two groups when U6 snRNA was used as an endogenous control for data normalization. However, when using miR-16 as an endogenous control, the relative levels of miR-106, let-7d and miR-21 before and after *H. pylori* eradication were significantly higher in the high-risk group than in the control group [31]. Another study focusing on suitable reference genes for qRT-PCR analysis of serum miRNAs indicated that miR-16 and miR-93 were the most stably expressed reference miRNA genes across patients and healthy controls [28]. Here, we also used different reference genes (U6 snRNA and miR-16) in miR-191 data analysis. The difference between miR-191 levels in the patient and healthy groups varied depending on whether U6 snRNA (*p* = 0.036) or miR-16 (*p* = 0.007) was used as a reference gene in our experiments.

Another study dealing with GC samples has demonstrated that miR-191 was upregulated in gastric carcinoma; however, the functional and mechanistic significance of miR-191 in gastric carcinogenesis was undefined. In this study, we demonstrated that reduced expression of miR-191 in the GC cell line HGC-27 resulted in impaired cell proliferation and inhibition of cell cycle progression. These results allow us to speculate that silencing of miR-191 may provide a survival advantage to GC cells. A similar oncogenic phenotype was also observed in HGC-27 cells with miR-425, another component originating from the same gene cluster as miR-191. This is consistent with the speculation that clustered genes often reinforce their functions in a common cellular pathway. Furthermore, as metastasis is the major cause of morbidity and mortality from GC patients, we subsequently analyzed the affects of miR-191 and miR-425 levels on cell migration and invasion. Inhibition of miR-191 and miR-425 both slowed HGC-27 cell migration in wound healing assays and suppressed cell invasion in transwell analysis, further confirming their association with the degree of GC malignance.

## 3. Experimental Section

### 3.1. Tissue Specimens

Gastric cancers and their associated morphologically normal tissue (located >3 cm away from the tumor) were obtained between November 2009 and November 2013 from 75 gastric cancer patients undergoing surgery at the Cancer Hospital of Chinese Academy of Medical Sciences (Beijing, China) (CICAMS, *n* = 22), Chinese PLA General Hospital (Beijing, China) (301 hospital, *n* = 33), and China-Japan Friendship Hospital (Beijing, China) (*n* = 20). Five normal gastric tissues from healthy people were obtained from gastroscopic biopsies at the China-Japan Friendship Hospital. Tissue samples were cut into two parts: one was fixed with 10% formalin for histopathological diagnosis, and the other was immediately snap-frozen in liquid nitrogen and stored at −196 °C in liquid nitrogen until RNA extraction. This group consisted of 65 males and 10 females with a median age of 57 years (range, 31–71 years). The use of the tissue samples for all experiments was approved by all the patients and by the Ethics Committee of the institution. The characteristics of patients included are described in Table S1.

### 3.2. Serum Collection

Whole-blood GC samples were collected from patients at the Cancer Hospital of Chinese Academy of Medical Sciences (CICAMS, *n* = 17), Chinese PLA General Hospital (301 hospital, *n* = 25) and China-Japan Friendship Hospital (*n* = 15). All blood samples were taken at the time of initial consultation before definitive surgical intervention and/or adjuvant therapy. Fifty-eight serum samples from healthy individuals (30 women, 28 men, median age 43) were used as controls. None of them had previously been diagnosed with a malignancy. Ethical permission and informed consent were obtained for the use of all samples. Blood samples were centrifuged at 3000 rpm for 10 min at 4 °C to completely remove cellular components, and the supernatant (serum) was collected. Then, the sera were immediately frozen at −80 °C until use.

### 3.3. Serum RNA Isolation and qRT-PCR

RNA was extracted from 500 μL of serum using QIAamp Circulating Nucleic Acid kits (Qiagen, Hilden, Germany), eluted into 60 μL of elution solution according to the manufacturer’s protocol, then stored at −80 °C until further processing. A volume of 20 μL was used for reverse transcriptase reactions, containing 12 μL of purified serum RNA, 4 μL 5× RT buffer, 1 μL of each dNTP (10 mM), 1 μL 200 U/μL M-MLV reverse transcriptase (Promega, Madison, WI, USA), 1 μL 40 U/μL RNase Inhibitor, 2 μL 1 M DTT, and 1 μL antisense looped primer. The mixture was incubated at 55 °C for 5 min, 72 °C for 60 min, and 85 °C for 5 min. Expression levels of miRNAs were analyzed by quantitative reverse-transcriptase PCR (qRT-PCR). Pre-designed TaqMan MicroRNA Assays including primer set and TaqMan probe were purchased from Applied Biosystems (Carlsbad, CA, USA). PCR was performed using TaqMan Universal PCR Master Mix II, and all reactions were performed in triplicate on the 7900HT Fast Real-Time PCR system (Applied Biosystems, Carlsbad, CA, USA). All PCR reactions, including no-template controls, were run in triplicate. The cycle passing threshold (*C*q) was recorded for each candidate miRNA, and U6 snRNA and miR-16 were used as endogenous controls for data normalization. Relative expression of the target miRNAs was calculated using the comparative threshold cycle method. The average coefficients of variation (%CV) for miR-191 intra-run and inter-run (3 runs total for 115 serum samples) were 1.96% ± 0.89% and 1.28% ± 0.60%, respectively. The average %CVs for miR-425 intra-run and inter-run were 1.70% ± 0.54% and 2.16% ± 0.55%, respectively.

### 3.4. Cell Cultures and Transfection

Three human gastric cancer cell lines were examined in this study: MGC-803 (mucinous gastric carcinoma, poorly differentiated), HGC-27 (metastatic lymph node, undifferentiated carcinoma), MKN-45 (Signet ring carcinoma, poorly differentiated) and SGC-7901 (adenocarcinoma, moderately differentiated). The MGC-803 cell line was purchased from the Cell Resource Center of Institute of Basic Medical Sciences, Chinese Academy of Medical Sciences and Peking Union Medical College (Beijing, China) and was propagated in Dulbecco’s modified Eagle medium (Gibco; Invitrogen; Life Technologies, Carlsbad, CA, USA), supplemented with 10% fetal bovine serum (FBS; PAA, Pasching, Austria), streptomycin (100 μg/mL) and penicillin (100 U/mL). HGC-27, MKN-45 and SGC-7901 cell lines were provided by American Type Culture Collection (ATCC; Manassas, VA, USA) and were maintained in RPMI 1640 medium (PAA) supplemented with 10% FBS (PAA). HGC-27 human gastric cancer cells were transfected with miRNA inhibitors (GenePharma; Shanghai, China), negative control miRNA (Scramble; GenePharma; Shanghai, China) at a final concentration of 25 nmol/L using DharmaFECT 1 (Dharmacon, Lafayette, CO, USA), in accordance with the manufacturer’s instructions.

### 3.5. Tissue RNA Isolation and qRT-PCR

Total RNA was extracted from the cells and tissues with TRIzol reagent (Invitrogen, Carlsbad, CA, USA). MicroRNAs were quantified by qRT-PCR using TaqMan MicroRNA assay (Invitrogen, Carlsbad, CA, USA). First-strand complementary DNA (cDNA) synthesis was carried out as described above. QRT-PCR was performed as described above, but with 1 μL of RT product. All RT reactions included no-template controls and were run in triplicate. The cycle passing threshold (*C*q) was recorded for candidate miRNAs, and miRNA quantification data were normalized to U6 (Table S2). The average %CVs for miR-191 intra-run and inter-run (6 runs total for 75 paired samples) were 1.51% ± 0.44% and 2.95% ± 0.87%, respectively. The average %CVs for miR-425 intra-run and inter-run were 2.08% ± 0.63% and 3.84% ± 1.21%, respectively.

### 3.6. Cell Proliferation and Cell Cycle Assay

Cells were incubated in 10% CCK-8 (DOJINDO, Fukuoka, Japan) diluted in normal culture medium at 37 °C until visual color conversion occurred. Proliferation rates were determined at 0, 24, 48, 72 and 96 h after transfection. The absorbance of each well was measured with a microplate reader set at 450 and 630 nM. All experiments were performed in quadruplicate.

Cell cycle analysis was performed on HGC-27 cells 48 h after transfection with either miRNA mimics or scramble control. Cells were harvested, washed twice with cold PBS, fixed in ice-cold 70% ethanol, incubated with propidium iodide (PI) and RNase A, and analyzed by FACS. Each sample was run in triplicate.

### 3.7. Cell Migration and Invasion Assays

HGC-27 cells were grown to confluence on 12-well plastic dishes and treated with miRNA mimics or scramble control. At 24 h post-transfection, linear scratch wounds (in triplicate) were created on the confluent cell monolayers using a 200 μL pipette tip. To remove cells from the cell cycle prior to wounding, cells were maintained in serum-free medium. To visualize migrated cells and wound healing, images were taken at 0, 24 and 48 h. A total of ten areas were selected randomly from each well, and the cells in three wells of each group were quantified.

For the invasion assays, after 24 h of transfection, 1 × 10^5^ cells in serum-free media were seeded onto the transwell migration chambers (8 μm pore size; Millipore, Darmstadt, Germany) which coated with the upper chamber of an insert coated with Matrigel (Sigma-Aldrich, St. Louis, MO, USA). Media containing 20% FBS was added to the lower chamber. After 24 h, the non-invading cells were removed with cotton wool, and invasive cells, located on the lower surface of the chamber, were stained with May-Grunwald-Giemsa stain (Sigma-Aldrich, St. Louis, MO, USA) and counted using a microscope (Olympus, Tokyo, Japan). Experiments were independently repeated three times.

### 3.8. Statistical Analyses

Student’s *t*-test (two-tailed) was performed to analyze the data. The analysis of variance (ANOVA) was used to determine whether there were any significant differences between groups. Receiver-operator characteristic curves (ROC) were obtained to establish the most suitable cutoff point. Mantel-Haenszel statistics were used to assess the relationship of serum levels of miRNAs between the GC group and the control group. A two-sided *p*-value of less than 0.05 was considered statistically significant. * *p* < 0.05; ** *p* < 0.01. All statistical computations were performed using SPSS (SPSS Inc.: Chicago, IL, USA).

## 4. Conclusions

Our study revealed two upregulated miRNAs, miR-191 and miR-425, in GC patients. Their oncogenic roles in gastric carcinogenesis were supported by their role in the regulation of cancer cell proliferation, migration and invasion. More importantly, increased levels of serum miR-191 can distinguish, with significant specificity and sensitivity, patients with GC from healthy controls. Our results provide new insights into GC-associated miRNAs and a firm basis for the further investigation of miRNAs as blood-based GC predictive and prognostic biomarkers.

## Supplementary Information

**Table S2.** MIQE Checklist [32].

Item to Check	Importance	Check List	Where in the Manuscript; Additional Comment
Experimental Design			

Definition of experimental and control groups	E	Yes	Materials and Methods: GC tissues and matched adjacent normal tissues; and in Table S1.
Number within each group	E	Yes	Materials and Methods: (1) 75 pairs of clinic GC tissues and matched adjacent normal tissue samples; (2) 57 GC patients and 58 healthy donor blood samples.
Assay carried out by core lab or investigator’s lab?	D	Yes	All assays were performed in investigator’s lab.
Acknowledgement of authors’ contributions	D	Yes	All mentioned authors met the authorship as defined by the journal.
SAMPLE			
Description	E	Yes	Materials and Methods.
Volume/mass of sample processed	D	Not available	
Microdissection or macrodissection	E	Yes	Materials and Methods: macrodissection with histolological verification.
Processing procedure	E	Yes	Materials and Methods.
If frozen—how and how quickly?/If fixed—with what, how quickly?	E	Yes	Materials and Methods: all samples were immediately snap-frozen in liquid nitrogen, and stored at −196 °C in liquid nitrogen until RNA extraction.
Nucleic Acid Extraction			
Procedure and/or instrumentation	E	Yes	Materials and Methods.
Details of DNase or RNAse treatment	E	Yes	No treatment.
Contamination assessment (DNA or RNA)	E	Yes	According to Chen *et al.* [ 33] miRNA measurements by the TaqMan assays are not affected by genomic DNA.
Nucleic acid quantification	E	Yes	The RNA was measured by Thermo Scientific™ Nanodrop™ 2000.
Reverse Transcription			
Complete reaction conditions	E	Yes	Materials and Methods.
Cqs with and without RT	D	Yes	There were no *C*qs (<40) in reactions without RT.
Storage conditions of cDNA	D	Yes	The cDNA was stored at −20 °C.
qPCR Target Information		Yes	
Sequence accession number	E	Yes	Materials and Methods: Predesigned TaqMan MicroRNA Assays including primer set and TaqMan probe were purchased from Applied Biosystems. The miRBase Accession Numbers for miR-191 (MIMAT0000440) and miR-425 (MIMAT0003393).
Location of amplicon/Amplicon length/In silico specificity screen (BLAST, *etc.*)/Pseudogenes, retropseudogenes or other homologs?/Secondary structure analysis of amplicon	E	Yes	Materials and Methods: Use of miRNA specific TaqMan assays; specificity guaranteed by the manufacturer.
qPCR Oligonucleotides			
Primer sequences/Probe sequences/Location and identity of any modifications	E	Yes	The manufacturer does not provide this information for miRNAs.
Manufacturer of oligonucleotides	D	Yes	Applied Biosystems as part of Life Technologies.
Purification method	D	Yes	Applied Biosystems does not provide information.
qPCR Protocol			
Complete reaction conditions	E	Yes	Materials and Methods.
Reaction volume and amount of cDNA/DNA/Primer, (probe), Mg++ and dNTP concentrations/Polymerase identity and concentration/Buffer/kit identity and manufacturer	E	Yes	Use of miRNA specific TaqMan assays; specificity guaranteed by the manufacturer.
Exact chemical constitution of the buffer	D	Yes	The manufacturer does not provide this information.
Additives (SYBR Green I, DMSO, *etc*.)	E	Yes	No additional additives.
Manufacturer of plates/tubes and catalog number	D	Yes	The MicroAmp® Optical 384-Well Reaction Plates (4343370) were purchased from Applied Biosystems.
Complete thermocycling parameters	E	Yes	Use of miRNA specific TaqMan assays; specificity guaranteed by the manufacturer.
Reaction setup (manual/robotic)	D	Yes	Manual setup.
Manufacturer of qPCR instrument	E	Yes	7900HT Fast Real-Time PCR system (Applied Biosystems).
qPCR Validation			
Evidence of optimisation (from gradients)	D	Not available	
Specificity (gel, sequence, melt, or digest)	E	Yes	Specificity guaranteed by the manufacturer of the TaqMan assays.
Calibration curves with slope and y-intercept	E	Not available	
DATA ANALYSIS			
qPCR analysis program (source, version)	E	Yes	Materials and Methods: Relative expression of the target miRNAs was calculated using the ΔΔCq method.
Results of NTCs	E	Yes	NTC did not result in any amplification (data not shown).
Justification of number and choice of reference genes	E	Yes	Materials and Methods: U6snRNA and miR-16 were used as the endogenous control for data normalization, respectively.
Description of normalisation method	E	Yes	Materials and Methods.
Number and stage (RT or qPCR) of technical replicates	E	Yes	Materials and Methods: All PCR reactions including no-template controls were run in triplicate.
Repeatability (intra-assay and inter-assay variation, %CV variation)	E	Yes	Materials and Methods.
Statistical methods for result significance	E	Yes	Materials and Methods.
Software (source, version)	E	Yes	Materials and Methods.
Cq or raw data submission using RDML	D	Not available	

(All essential information (E) must be submitted with the manuscript. Desirable information (D) should be submitted if available).
